# Application of a Tensile Test Method to Identify the Ductile-Brittle Transition of Caramel

**DOI:** 10.3390/foods11203218

**Published:** 2022-10-14

**Authors:** Dennis Schab, Lydia Tiedemann, Harald Rohm, Susann Zahn

**Affiliations:** Chair of Food Engineering, Institute of Natural Materials Technology, Technische Universität Dresden, 01062 Dresden, Germany

**Keywords:** tensile test, viscoelasticity, fracture, stress, strain, cutting

## Abstract

During cutting of foods, tensile stresses in front of the blade are responsible for the separation of the material. Therefore, tensile tests can be helpful to gain knowledge on deformation properties related to pre-fracture cutting behavior as well as on phenomena in the fracture zone, which are velocity-dependent in viscoelastic materials. The aim of this work was to apply a tensile test method for model caramels to investigate their behavior and to identify conditions where the ductile-brittle transition occurs. After executing pre-trials, tensile velocity, caramel moisture, and temperature were the parameters that were varied for this purpose. In general, increasing velocity, decreasing temperature, and decreasing moisture resulted in a stiffer response and caused a shift from a ductile to a more brittle behavior, attributable to reduced viscous contributions to the material and longer relaxation times. Fracture strain was notably lower than the maximum plastic elongation in the ductile case, but we observed equalization close to the ductile-brittle transition point for our material. This study serves as basis for an in-depth research on the complex deformation and fracture phenomena during cutting of viscoelastic food systems, including numerical modeling.

## 1. Introduction

Most foods are relatively soft and exhibit viscoelastic behavior, showing both elastic, reversible deformation and viscous energy dissipation upon load. The latter is responsible for a time-dependent behavior expressed as relaxation or creep, which leads to stress reduction in the material and enables it to be further deformed in a ductile manner. When the time scale is too short for viscous energy dissipation, which sufficiently compensates for the build-up of stresses during loading, the result is brittle fracture that occurs when the strength of the material is exceeded [1,2]. Therefore, ductile failure of a viscoelastic material often happens only after large plastic deformation whereas brittle fracture is associated with little or no irreversible deformation [3].

According to van Vliet et al. [4], the energy required for material separation comprises work for elastic and for viscous deformation but also some dissipative work for the fracture process itself, namely for overcoming material cohesion and for creating new surfaces. The latter is released from the elastically stored energy [5,6] and is represented by a material parameter denoted as fracture toughness, which is velocity-dependent in case of viscoelastic materials. If viscous energy dissipation is the dominating fraction of the total energy needed for the deformation process, fracture toughness will decrease with increasing separation velocity because the reduced time scale inhibits dissipative processes in the material [2,7]. Consequently, ductile material failure is usually associated with a higher overall energy requirement than brittle failure.

Fracture substantially comprises three phases: crack initiation is followed by crack propagation and, finally, by failure of the work piece [8]. As for most materials, foods contain inhomogeneities, for instance, fine air bubbles that act as stress concentrators upon deformation [9]. If local stresses exceed fracture toughness, these inhomogeneities serve as starting point for cracks from which they propagate through the material. However, crack initiation is affected by the ratio of elastically stored to dissipated energy as well as by the energy input rate. If deformation velocity is too low, too much of the energy needed for fracture to occur will dissipate [1,2,3,5]. Generally, three fundamental modes of fracture can be distinguished. Mode I is characterized by crack opening due to tensile stresses whereas in modes II and III, material separation is caused by shear stresses acting normal or parallel to the crack front, respectively [10]. The predominant fracture mode that occurs in practice is mode I [11], which also applies for food processing, e.g., conventional straight edge blade cutting [12,13].

Cutting is the major industrial process for separating foods into pieces of defined shape and size. Penetrating and driving through a material with a blade initiates complex interactions between deformation and fracture phenomena but also friction forces, which result from the shearing motion between the blade and the material’s surface [14,15]. The separation induced by blade motion is caused by tensile stresses perpendicular to the cutting plane. In this context, finite element analysis was used to demonstrate that fracture stress determined in uniaxial tensile tests is a good indicator for predicting the onset of cutting in elastomeric materials [13]. When viscous properties significantly contribute to material behavior, as it is the case in real foods, the velocity dependency of the maximum stress inducing the cut must also be taken into account [2,12].

Tensile tests were already used for evaluating deformation and fracture behavior of potato starch gels [16], gellan gum gels [17], melted cheese [18], Mozzarella [19], chewy candy [20], toffee [21] and cabbage [22]. As concerns cutting, tensile tests can also be used for obtaining knowledge on velocity-dependent deformation properties linked to pre-fracture cutting behavior. In this context, studies on elastomers [13] or synthetic viscoelastic food models [12] were performed, but there is still a lack of research with real food systems.

The aim of this study was therefore to establish a tensile test method for determining rate-dependent deformation and fracture properties of a viscoelastic model food with special emphasis on the brittle-ductile transition. We previously demonstrated this transition for model caramels in cutting tests [23] and, following that work, again chose caramel as a test material. Caramel refers to a food comprised of an amorphous sugar matrix as continuous phase and sucrose crystals, a protein network, fat droplets, and fine air bubbles that are dispersed throughout the material. Caramel exhibits either a ductile, steady state cutting behavior or a pronounced brittle behavior with crack propagation preceding the blade tip when cut with low or high velocity, respectively [2,23]. Apart from cutting velocity, the final moisture content of the caramel has been identified as the most critical factor influencing the material’s response. In this study, the impact of moisture content, testing velocity, and temperature on the tensile behavior of caramel was investigated. The results shall provide a basis for future work concerning the role of tensile stresses in the complex cutting process of real foods, as well as for developing a numerical model describing the material’s behavior.

## 2. Materials and Methods

### 2.1. Caramel Preparation

The formulation of the caramel premix comprised 29.4% (*w*/*w)* granulated sucrose (Südzucker AG, Mannheim, Germany), 29.4% glucose syrup (45 °Bé; Grafschafter Krautfabrik Josef Schmitz KG, Meckenheim, Germany), 24.4% deionized water, 8.0% skim milk powder (Sachsenmilch Leppersdorf GmbH, Wachau, Germany), 7.9% fat (Bavettin 22830/SG palm oil from Walter Rau Neusser Öl und Fett AG, Neuss, Germany and Palmetta 70560/SG palm stearin from Bressmer & Francke GmbH & Co. KG, Norderstedt, Germany in equal shares), and 0.9% sunflower lecithin (Cargill s.r.l., Padova, Italy).

All products were cooked in a stainless steel vessel placed on a CT2010/IN induction heater (Rommelsbacher ElektroHausgeräte GmbH, Dinkelsbühl, Germany) in 610 g batches following Schab et al. [23]. After mixing all ingredients with an inclined-blade stirrer mounted on a Eurostar 60 device (IKA-Werke GmbH & Co. KG, Staufen, Germany) for 1 min at 250 rpm, heater power was set to 200 W. Caramel temperature was continuously monitored by a thermocouple and a 175-T3 temperature logger (Testo SE & Co. KGaA, Titisee-Neustadt, Germany). When all components were liquefied or dissolved, stirrer speed was increased to 700 rpm to homogenize the mass, and set back to 250 rpm once caramel temperature reached 90 °C. Above 100 °C, stirring was superseded by manually scraping the bottom of the vessel with a heat-proof plastic scraper. To achieve a final moisture in the range of approx. 8.0–6.5 g/100 g, cooking end temperature was 119 °C, 120 °C or 122 °C. To remove bulk heat, the vessel was subsequently placed in ice water for approx. 10 s.

**Figure 1:**
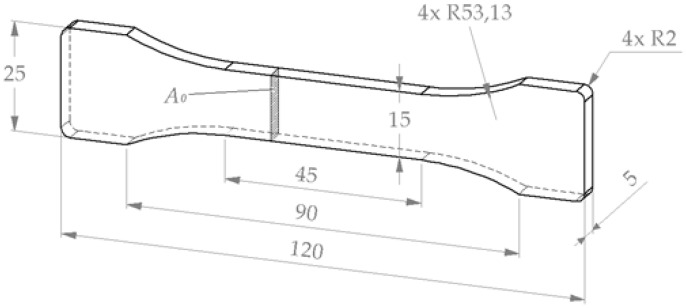
3D-drawing of a dogbone-shaped specimen. Dimensions are in mm.

Using plastic syringes with cut-off tips, the caramel mass was quickly poured into PTFE molds with dogbone-shaped recesses; specimen dimensions are given in Figure 1. The molds were then covered using silicone mats and kept in zip-lock bags for approx. 24 h at room temperature. Small parts of the caramel were kept in box-shaped containers.

### 2.2. Moisture Analysis

After filling grated caramel into pre-dried glass dishes, moisture content was determined in triplicate by drying at 60 °C and < 2 kPa in a VT 6060 M vacuum dryer (Thermo Fisher Scientific, Waltham, MA, USA) until mass constancy.

### 2.3. Execution of Tensile Tests

Prior to tensile testing, excess material protruding from the PTFE molds was slightly warmed using a hot-air gun and removed using a craft knife. The specimens were then removed from the molds and kept in an incubator at 18 °C, 25 °C or 32 °C for approx. 2 h.

Tensile tests were performed using a 5564 universal testing machine (Instron GmbH, Darmstadt, Germany) equipped with a 1 kN force transducer. At all crosshead velocities *v* that ranged between 100 mm/min and 2500 mm/min, force and displacement signals were collected using an NI-9215 A/D-converter (National Instruments, Austin, TX, USA) at a rate of 60 data points per mm deformation. Appropriate calibration functions were used to convert the respective voltage signals into force or deformation units.

The specimens were slightly fixed in tensile clamps covered with emery paper and pre-strained by applying approx. 0.5 mm deformation. Proper alignment of the specimens in the clamps was checked with a self-leveling cross line laser (Quigo green, Bosch GmbH, Stuttgart, Germany). Subsequently, specimens were tightened between the clamps at 0.5 N.m using a 7440 ESD torque wrench (Wera Werkzeuge, Wuppertal, Germany). Effective clamping distance was either 90 mm or, after initial experiments, 45 mm (see Figure 1). Tensile tests were stopped either after fracture of the specimens or after reaching 65% strain and conducted in triplicate.

### 2.4. Data Calculatiuon

Force/deformation curves data were smoothed using a sliding average over 6 data points, corresponding to an absolute distance of 0.1 mm. Engineering strain *ε* was calculated by relating displacement *u* to initial between-clamp length *l*_0_, and engineering stress *σ* by relating force *F* to initial cross-section *A*_0_ of the sample, i.e., 75 mm^2^ (see Figure 1).

The modulus *E*, corresponding to the initial slope of the *σ*/*ε* function, was obtained by applying linear regression [24]. Fracture stress *σ*_f_ and tensile strength *R*_m_ refer to the stress maximum of either fractured, brittle or of non-fractured, ductile specimens, respectively. By considering the modulus, we further determined fracture strain *ε*_f_, or maximum plastic elongation *ε*_p_ for the non-fractured specimens.

### 2.5. Data Treatment

All data are presented as arithmetic mean ± standard deviation. The significance of differences between means was estimated using one-factor analysis of variance and Tukey post-hoc tests (Origin Lab Corporation, Northampton, MA, USA).

## 3. Results and Discussion

### 3.1. Preliminary Screening Trials

**Figure 2:**
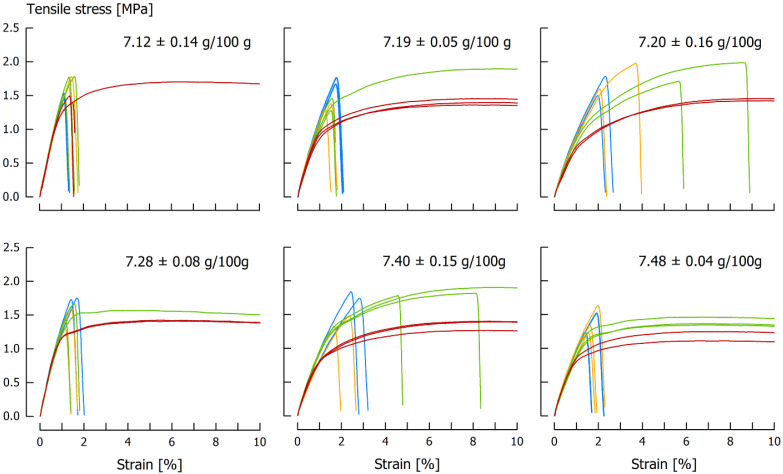
Tensile stress/strain curves of six caramel samples (*T* = 25 °C) tested at *v* = 1000 (red), *v* = 1375 (green), *v* = 1750 (yellow) and *v* = 2500 mm/min (blue). Moisture content of the samples is given in the charts. For the sake of clarity, curves from the *v* = 2125 mm/min are not displayed.

The first set of experiments was performed to obtain information on the range of tensile velocities that need to be applied to identify the transition point from ductile to brittle behavior. For this purpose, six caramel batches (*T* = 25 °C) were tested at a clamping distance of 90 mm and at a tensile velocity of 1000, 1375, 1750, 2125 or 2500 mm/min (Figure 2). The unique end temperature during cooking of 120 °C resulted in a caramel moisture that ranged between 7.12 g/100 g and 7.48 g/100 g. At the lowest velocity, all samples showed ductile behavior, and a tendency toward a higher plateau force at lower caramel moisture is evident. A few specimens started to fracture at *v* = 1375 mm/min and, for *v* = 1750 mm/min or higher, all samples fractured.

**Figure 3:**
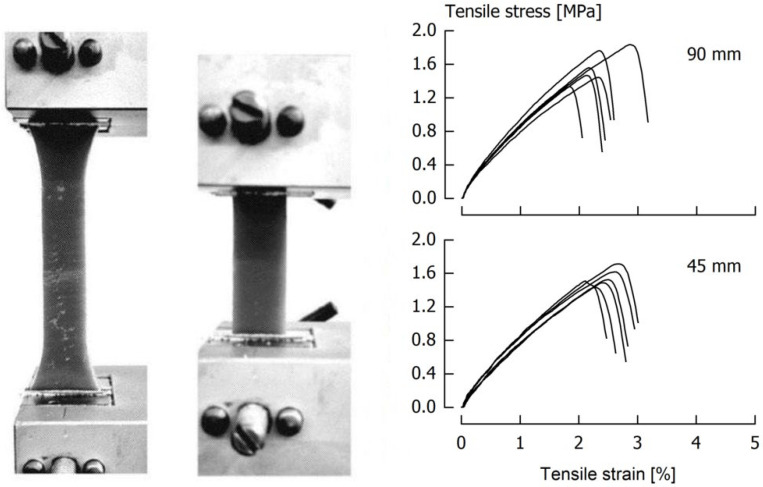
Tensile clamps with specimens fixed at a clamping distance of 90 mm or 45 mm, and the resulting stress/strain curves of repeated tests performed at *v* = 2500 mm/min.

However, during execution of the tests and when checking the specimens after testing, we observed that approximately one third fractured either in the necking position or near the clamps. Such a behavior can be attributed to a concentration of local stresses in the region where sample width becomes larger [17], which causes cracks to start at such locations [25]. As, according to ISO [24], specimens that either slip in the tension clamps or that fracture in the necking region at undefined cross-section should be eliminated from further data processing, it was decided to test the procedure against specimens that were fixed in the clamps at a distance of 45 mm (Figure 3). This way of gripping dogbone-shaped specimens has been demonstrated, for instance, by Ma et al. [26] and by McCulloch [27]. It is evident from the resulting stress/strain curves of one of three sets of experiments, performed on a caramel with 7.15 ± 0.02 g/100 g moisture and at *v* = 2500 mm/min, that clamping at 45 mm results in a fracture stress of 1.54 ± 0.11 MPa that is insignificantly (*p* > 0.05) different from *σ*_f_ = 1.56 ± 0.19 MPa obtained for clamping at 90 mm. As a lower coefficient of variation for the 45 mm clamping procedure was also obtained for another two sample sets and for fracture strain, it was decided to test a reduced clamping distance of 45 mm at lower tensile velocities.

For this purpose, three individual sets of caramel (moisture content 7.19 g/100 g, 7.20 g/100 g and 7.28 g/100 g) were each split in three parts and tested at three different conditions, either at *v* = 1000 mm/min or *v* = 2500 mm/min. Comparing an effective specimen length of 90 mm and 45 mm means that also the extension rate $\dot{\varepsilon }$ at any velocity, calculated as *v*/*l*_0_, differs by a factor of 2. The reduction of *l*_0_ from 90 mm to 45 mm, resulting in an increase of $\dot{\varepsilon }$ from 11.1/min to 22.2/min when testing at *v* = 1000 mm/min, was responsible for a different behavior of the caramel (Table 1). Whereas samples with *l*_0_ = 90 mm tested at *v* = 1000 mm/min (condition A) proved to be ductile with a tensile strength of 1.42 ± 0.02 MPa, all specimens fractured when the extension rate was higher because of the reduced initial specimen length; in this case (condition B: *l*_0_ = 45 mm, *v* = 1000 mm/min), *σ*_f_ was 1.66 ± 0.07 MPa (*p* < 0.05). When the higher strain rate was achieved by applying a velocity of *v* = 2500 mm/min on longer specimens (condition C), fracture stress was on a similar level (1.69 ± 0.05 MPa).

### 3.2. Velocity Dependency

**Figure 4:**
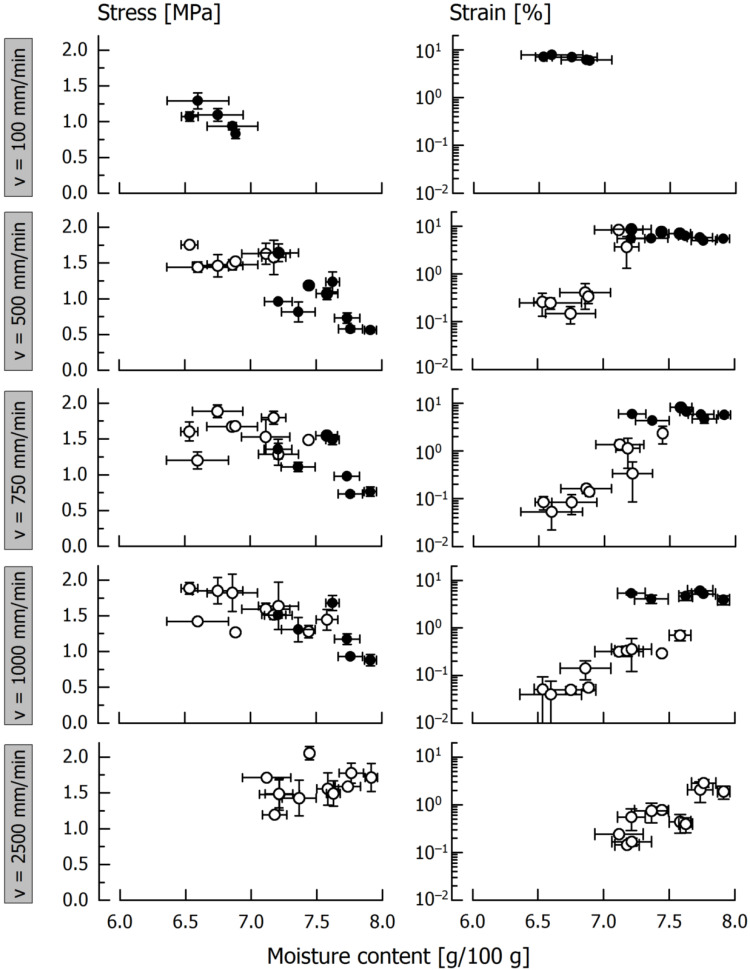
Fracture stress *σ*_f_ (open circles) and tensile strength *R*_m_ (closed circles) as well as fracture strain *ε*_f_ (open circles) and maximum plastic elongation *ε*_p_ (closed circles) of caramels subjected to tensile testing at different velocity. Data are arithmetic means ± standard deviation for fracture stress or tensile strength and the corresponding strain (*n* = 3), and moisture (*n* = 3).

Figure 4 shows either fracture stress or tensile strength and fracture strain or maximum plastic deformation of caramels (*T* = 25 °C) with a moisture content between 6.5 g/100 g and 8.0 g/100 g obtained during testing at different tensile velocity. Individual caramel batches were kept relatively small so that it was only possible to produce 12 dogbone-shaped specimens per caramel cooking procedure. This was done because of two reasons: (1) to keep the cooking procedure (~60 min per batch) as well as the molding of specimens within a practical time frame and (2) to ensure that all specimens can be equally well molded (as viscosity rapidly increases when the mass cools down) and to minimize any possible effects of ongoing moisture evaporation. It was therefore decided to apply the lowest velocity (*v* = 100 mm/min) and to exclude the highest velocity (*v* = 2500 mm/min) only to samples with <7.0 g/100 g moisture whereas, at higher moisture, tests at *v* = 100 mm/min were not performed as a ductile behavior was taken for granted at this velocity. Consequently, three replicate tensile tests could be performed at each of the four selected velocities.

As regards *σ*_f_ or *R*_m_, and up to *v* = 1000 mm/min, we observed a significant decrease of either stiffness parameter with increasing moisture content. At the lowest tensile velocity of *v* = 100 mm/min, all samples showed ductile behavior. At *v* = 500 mm/min, the transition from ductile to brittle occurred when caramel moisture was lower than approx. 7.2 g/100 g. With further increasing velocity, this transition shifted toward higher moisture content and, at *v* = 2500 mm/min, all specimens fractured during testing. At this tensile velocity, the dependency of *σ*_f_ on caramel moisture was no more observable.

In case of the deformation measure, *ε*_p_ reached 10% at maximum in ductile samples, and we observed a slight trend toward decreasing *ε*_p_ with increasing moisture content especially at lower velocity. In case of brittle samples *ε*_f_ was, in most cases, < 1% and only exceeded this value in individual samples with the highest moisture content tested at the respective velocity. It is also evident from the log scale chart in Figure 4 that, at a given *v*, specimens fracture at a lower strain when caramel moisture is lower.

The critical stresses (*σ*_f_ or *R*_m_) in the individual experiments that build the basis of Figure 4 were subsequently used to calculate two-parameter Weibull distributions. As stated by Rojo and Vincent [28], an advantage of the Weibull model is that it allows to account for the complexity in fracture under a statistical approach that still yields fundamental material properties and that defines the probability of fracture *P*_f_(*σ*) by statistical estimators [8]. In the Weibull function

(1) $$P_{f}\left(\sigma \right)=1-exp\left[-{\left(\frac{\sigma }{\sigma_{0}}\right)}^{m_{W}}\right]$$

*P*_f_(*σ*) is a function of the observed fracture parameter *σ*, in the context of this study either *σ*_f_ or R_m_, and *σ*_0_ the characteristic stress of the material that refers to the stress at which *P*_f_(*σ*) = 1 − 1/e = 0.632. Consequently, 63.2% of the samples are expected to fracture or to approach maximum tensile strength when *σ*/*σ*_0_ = 1. The dimensionless Weibull modulus *m*_W_ describes the variability in material strength, with a lower *m*_W_ indicating a higher variability and a broader range of the ductile-brittle transition [29]. This parameter is estimated by linear regression after having assigned fracture probabilities to all the measured critical stresses using an estimator [30]. In context with fracture of foods the Weibull distribution was, for instance, used for uncooked pasta [31], dry-cured ham [32], roasted wheat kernels [33], peas [34] and potato crips [28].

**Figure 5:**
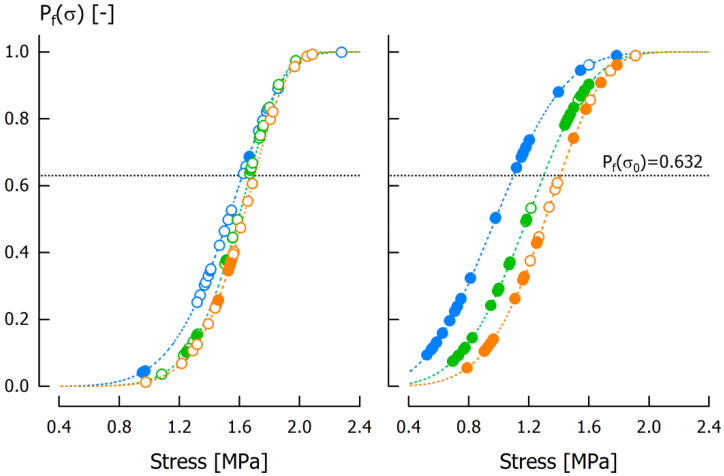
Weibull distribution probabilities *P*_f_(*σ*) based on caramel samples with low (6.53–7.20 g/100 g, **left**) and high moisture content (7.22–7.91 g/100 g, **right**). Stress on the abscissa refers to fracture stress *σ*_f_ (open circles) or tensile strength *R*_m_ (closed circles). Tensile velocity was *v* = 500 mm/min (blue), *v* = 750 mm/min (green) and *v* = 1000 mm/min (orange).

Before calculating the Weibull parameters, we separated our caramel sample set using the moisture median of 7.21 g/100 g, and then used the step-by-step approach outlined by Łysiak [34] to obtain *σ*_0_ and *m*_W_ individually for the tensile velocities that were applied to all samples (i.e., 500 mm/min, 750 mm/min, and 1000 mm/min). In case of a ductile response, the maximum tensile strength can be considered as the onset of ductile failure [35]. Hence, we treated both *σ*_f_ and *R*_m_ values equally in the calculation. The respective distributions of *P*_f_(*σ*) versus *σ* are displayed in Figure 5. For the caramel subset with low moisture, the characteristic stress *σ*_0_ was almost independent of *v* (Table 2). In addition, *m*_W_ was almost similar for *v* = 750 mm/min and *v* = 1000 mm/min, resulting in nearly identical probability distributions. Apart from the fact that the majority of the specimens fractured, the results indicate that tensile velocity has no impact on maximum material strength (i.e., *σ*_f_ or R_m_). This is likely due to the fact that there are less pronounced viscous contributions to the material’s behavior in low moisture samples, which also means that deformation until failure happens almost exclusively in the linear elastic region. The situation is, however, different for the caramel subset with higher moisture content (7.21–7.91 g/100 g). Both *σ*_0_ and *m*_W_ were lower than for the low moisture sample subset, meaning that the point of failure (now mostly ductile) is reached at lower stress and that the ductile-brittle transition region becomes broader. The more pronounced discrimination of the Weibull distributions comes from the enhanced velocity dependency of materials with higher amounts of viscous contributions [36]. Within the high moisture subset, both *σ*_0_ and *m*_W_ increased with increasing *v*. This increase is responsible for a horizontal shift to the right and a steeper probability distribution. In addition, the number of specimens that showed distinct fracture (open symbols refer to *σ*_f_ in Figure 5) increased with increasing tensile velocity. However, it should be considered that the Weibull distributions are based on the assignment of probabilities to the measured stresses by a statistical estimator so that a validation is only possible when large sample sets are subjected to tensile testing. According to Quinn & Quinn [29] a minimum of approximately 30 specimens provide an adequate Weibull distribution.

Our experiments clearly demonstrate that an increasing testing velocity results in a stiffer material response and a more brittle behavior, indicated by a lower fracture strain, of the caramel specimens at a distinct moisture. This also means that the brittle-ductile transition is shifted toward samples with higher moisture content which fits to our previous observations from cutting tests [23]. Moisture, on the other hand, has an analogous but counteracting effect, with increasing moisture leading to a more ductile behavior with significantly lower stresses and higher strains. It is interesting to note that within the brittle state, increasing velocity or moisture content only seems to affect fracture strain but not the fracture stress, with moisture having the more significant impact.

### 3.3. Temperature Dependency

**Figure 6:**
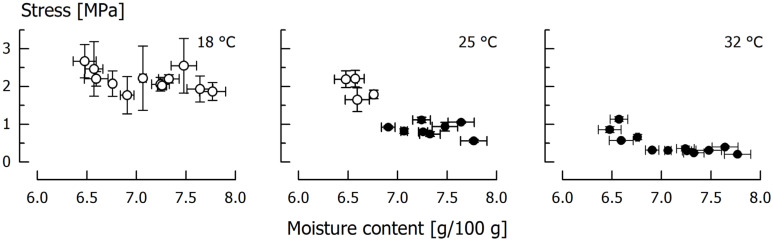
Fracture stress *σ*_f_ (open circles) and tensile strength *R*_m_ (closed circles) of caramel subjected to tensile testing at different temperature and at *v* = 1000 mm/min. Data are arithmetic means ± standard deviation for *σ*_f_ or *R*_m_ (*n* = 3), and moisture (*n* = 3).

Critical tensile stresses obtained through testing at different temperature but at a constant *v* = 1000 mm/min are shown in Figure 6. These tests were carried out using 12 batches of caramel with a moisture content ranging between 6.48 g/100 g and 7.77 g/100 g, similar to the velocity dependency experiments. Although performed on a different set of samples, the distribution of the tensile stresses at 25 °C is similar to the data distribution observed in the velocity experiments at *v* = 1000 mm/min (see Figure 4) which indicates a sufficient reproducibility.

Similar to applying a lower velocity, testing at a higher temperature also leads to a decrease of critical stress. This is because a higher temperature enhances molecular mobility by increasing intermolecular distances. By this way, the amorphous matrix becomes softer and rearrangement processes are facilitated [20,37]. At 18 °C, brittle fracture occurred for all caramels and a clear dependency of *σ*_f_ on caramel moisture was not observed. This is similar to experiments executed at the highest velocity but at 25 °C (see Figure 4). At a temperature of 25 °C, the brittle-ductile transition was detected for the given velocity at a moisture content of approximately 6.8 g/100g. As compared to Figure 4, there is no overlapping of ductile and brittle batches and the transition moisture seems to be slightly shifted which is probably just a result of the smaller sample size. Finally, at 32 °C, all samples behaved ductile with rather low tensile stresses. For both 25 °C and 32 °C, there is a significant (*p* < 0.005) linear correlation between the critical stress and moisture content (*r* = 0.84 and *r* = 0.77, respectively). At 25 °C, the average *R*_m_ of the ductile samples (moisture content > 6.9 g/100 g) was 0.87 ± 0.17 MPa. For the same samples measured at 32 °C, *R*_m_ was significantly lower (0.30 ± 0.06 MPa, *p* < 0.05). All of these samples fractured at 18 °C, with *σ*_f_ being 2.08 ± 0.25 MPa (*p* < 0.05).

**Figure 7:**
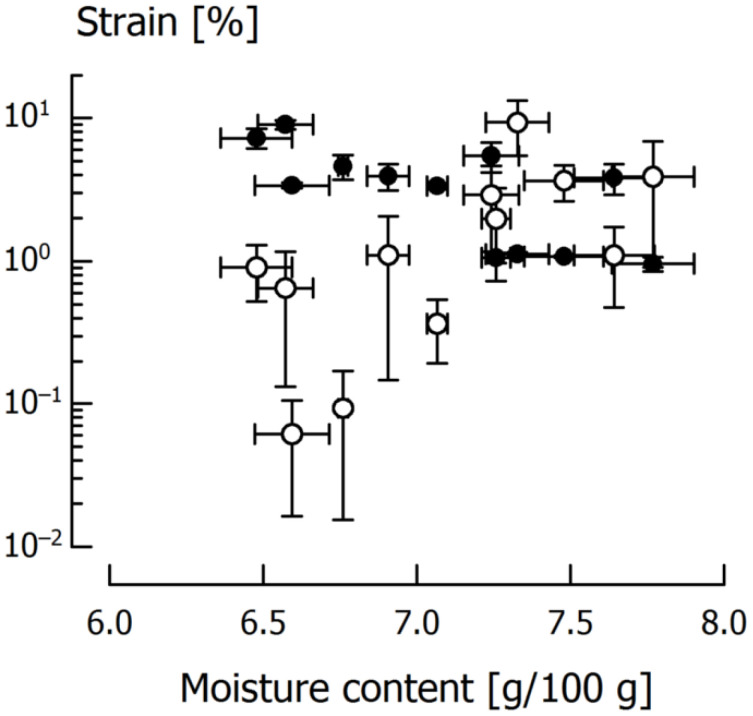
Fracture strain *ε*_f_ (open circles) and maximum plastic elongation *ε*_p_ (closed circles) of caramels subjected to tensile testing at 18 °C (all samples showed brittle fracture) and 32 °C (all samples showed ductile behavior), respectively, at *v* = 1000 mm/min. Data are arithmetic means ± standard deviation for *ε*_f_ or *ε*_p_ (*n* = 3), and moisture (*n* = 3).

For the tensile tests performed at 18 °C or 32 °C, the respective *ε*_f_ and *ε*_p_ are displayed in Figure 7, where the results at 25 °C are left out for the sake of clarity. Regarding increasing moisture content, there is a tendency for samples that undergo brittle fracture to fail at higher strains, and for ductile samples to fail at lower strains. This was likewise observed for tests with varying tensile velocity (see Figure 4). The temperature effect was insignificant.

Generally speaking, the results suggest that the strain that is associated with maximum material strength shows a maximum when the material’s behavior changes from brittle to ductile, independent of whether this is induced by velocity, temperature or, for our material, moisture content. This means that, as rearrangement processes are favored and relaxation times become shorter, the fracture strain increases, supposedly up until brittle-ductile transition is reached. From this point onward, the strain at maximum tensile strength decreases again because relaxation times are still shortening and the linear elastic region is exceeded after smaller deformation.

## 4. Conclusions

In this paper we applied a tensile test method to investigate the material properties and the ductile-brittle transition of viscoelastic foods for which we used caramel as a respective model [23]. As expected for viscoelastic materials, we observed a marked velocity dependency, with higher strain rates generally leading to higher tensile stresses. The same tendencies were observed when temperature or moisture content of the caramel decreased. For each of these parameters, the ductile-brittle transition could be shown when the other parameters were held constant. It was also possible to use Weibull statistics for demonstrating the effects of testing velocity on the shift from ductile to brittle behavior.

Future research using this work as a basis should focus more deeply on the connection between the material’s behavior in tensile tests and the complex phenomena in the fracture zone during cutting. The tensile test method should also lay the foundation for parameter identification concerning the development of a material model that can be used to numerically describe deformation and fracture behavior.

## Figures and Tables

**Table 1:** Effects of specimen length and tensile velocity on fracture properties (*T* = 25 °C).

	Condition A	Condition B	Condition C
Length *l*_0_	90	45	90
Tensile velocity [mm/min]	1000	1000	2500
Extension rate [1/mm]	11.1	22.2	27.8
Fracture stress [MPa] ^1^	-	1.66 ^b^ ± 0.07	1.69 ^b^ ± 0.07
Tensile strength [MPa] ^1^	1.42 ^a^ ± 0.02	-	

Mean values ± standard deviations (*n* = 12) in the lines with fracture stress and tensile strength marked by different superscripts differ significantly (*p* < 0.05).

**Table 2:** Effects of specimen length and tensile velocity on fracture properties (*T* = 25 °C).

Tensile Velocity [mm/min]	6.53–7.20 g/100 g Moisture		7.22–7.91 g/100 g Moisture	
	*σ*_0_ [MPa]	*m*_W_ [–]	*σ*_0_ [MPa]	*m*_W_ [–]
500	1.62	5.93	1.10	3.12
750	1.67	7.64	1.30	4.07
1000	1.70	7.88	1.41	4.96

## Data Availability

The data presented in this study are available on request from the corresponding author.
